# Impact of Using a 3D Visual Metaphor Serious Game to Teach History-Taking Content to Medical Students: Longitudinal Mixed Methods Pilot Study

**DOI:** 10.2196/13748

**Published:** 2019-09-26

**Authors:** Hussain Alyami, Mohammed Alawami, Mataroria Lyndon, Mohsen Alyami, Christin Coomarasamy, Marcus Henning, Andrew Hill, Frederick Sundram

**Affiliations:** 1 South Auckland Clinical Campus Faculty of Medical and Health Sciences The University of Auckland Auckland New Zealand; 2 College of Medicine Taif University Taif Saudi Arabia; 3 Auckland City Hospital Auckland New Zealand; 4 Middlemore Hospital Auckland New Zealand

**Keywords:** video games, instructional technology, memory, retention, metaphor, learning, clinical competence

## Abstract

**Background:**

History taking is a key component of clinical practice; however, this skill is often poorly performed by students and doctors.

**Objective:**

The study aimed to determine whether Metaphoria, a 3D serious game (SG), is superior to another electronic medium (PDF text file) in learning the history-taking content of a single organ system (cardiac).

**Methods:**

In 2015, a longitudinal mixed methods (quantitative and qualitative) pilot study was conducted over multiple sampling time points (10 weeks) on a group of undergraduate medical students at The University of Auckland Medical School, New Zealand. Assessors involved in the study were blinded to group allocation. From an initial sample of 83, a total of 46 medical students were recruited. Participants were assigned to either a PDF group (n=19) or a game group (n=27). In total, 1 participant left the PDF group after allocation was revealed and was excluded. A total of 24 students in the game group and 14 students in the PDF group completed follow-up 7 weeks later. Using an iterative design process for over a year, with input from a variety of clinical disciplines, a cardiac history-taking game and PDF file were designed and informed by Cognitive Load Theory. Each group completed its intervention in 40 min. A total of 3 levels of Kirkpatrick training evaluation model were examined using validated questionnaires: affective (perception and satisfaction), cognitive (knowledge gains and cognitive load), and behavioral attitudes (Objective Structured Clinical Exam) as well as qualitative assessment. A priori hypotheses were formulated before data collection.

**Results:**

Compared with baseline, both groups showed significant improvement in knowledge and self-efficacy longitudinally (*P*<.001). Apart from the game group having a statistically significant difference in terms of satisfaction (*P*<.001), there were no significant differences between groups in knowledge gain, self-efficacy, cognitive load, ease of use, acceptability, or objective structured clinical examination scores. However, qualitative findings indicated that the game was more engaging and enjoyable, and it served as a visual aid compared with the PDF file.

**Conclusions:**

Students favored learning through utilization of an SG with regard to cardiac history taking. This may be relevant to other areas of medicine, and this highlights the importance of innovative methods of teaching the next generation of medical students.

## Introduction

### Background

Since the time of Hippocrates, clinical history taking has remained a cornerstone of medicine [1]. Although history taking is the most common medical procedure performed by doctors [2], research has shown a deficit in this essential skill among medical students [3-6], interns, and, more worryingly, general practitioners [7]. History taking is more valuable than physical examination in reaching a diagnosis in around 80% of medical outpatient referrals [8,9]. When adequately performed, history taking is associated with improved patient physiological and psychological health outcomes [10], satisfaction [11,12], and compliance [13,14]. Although the literature shows that this essential clinical skill can be learned, it is inadequately taught [3,5,15].

A fine balance between the history-taking components, that is, history-taking content (HTC) and history-taking process (HTP), needs to be achieved for optimal effectiveness. HTC is commonly referred to as data or information gathering, and it is concerned with the *what* part of the medical interview, eliciting specific information about the patient’s symptomatology from the presenting complaint through to social and occupational histories [1]. On the other hand, HTP, commonly known as communication skills, is the method by which this content is elicited; thus, it is more concerned with the *how* of the medical interview [1]. There is evidence to suggest that medical students are distracted when trying to remember HTC, which impairs their communication skills [16,17]. Despite the importance of teaching HTC, this remains an understudied area, with a recent systematic review showing only 6 studies of educational interventions that targeted this essential part of the medical interview; this review also found that technologically enhanced interventions improved teaching of HTC [18].

### Objectives

The literature shows that medical students use multiple learning styles, including visual, auditory, and kinesthetic [19]; therefore, we propose that a visual metaphor system could prove to be of significant value to enhance the teaching of HTC. A visual metaphor is defined as “a graphic structure that uses the shape and elements of a familiar natural or man-made artifact or of an easily recognizable activity or story, to organize content meaningfully and use the associations with the metaphor to convey additional meaning about the content” [20:233]. The use of visual metaphors in medicine dates back to antiquity, when Aristotle explained how blood is contained in the heart and associated vessels and compared this with a “vase” [21]. Currently, visual metaphors continue to be used successfully to teach some of the more complicated and abstract scientific principles, for example, Emil Fischer’s Lock-and-Key model of enzyme-substrate interaction in physiology [22]. Until recently, metaphor was considered a decorative component of speech; it is now considered “to pervade all forms of knowledge” [23:86]. A number of benefits have been reported for using a visual metaphor system as a learning and knowledge sharing tool, including improved audience engagement, attention, memory, and comprehension [24]. Visual metaphors can provide a means of transferring a complex concept by using simple visual symbols that facilitate the connection between thoughts and feelings [25]. Visual metaphors, when created and presented well, enable high levels of content comprehension, retention, and recall, and they are easier to construct and interpret, compared with mind maps [20]. They also assist learners to incorporate newly learned material with previous knowledge [26,27]. Serious games (SGs) offer a promising modality of delivering visual metaphors, as they have been shown to provide learners with an “anchor” for knowledge [28]. Fabricatore described how metaphors could be embedded in SGs to enhance learning, especially if they are designed to being at the heart of gameplay rather than serving as a mere decorative component [29]. There are potential benefits of SGs, which utilize visual metaphorical elements, in medical education [30]; however, the application and assessment of metaphor use in SG design are limited [31]. SGs are defined as games “that engage the user and contribute to the achievement of a defined purpose other than pure entertainment” [30: 5]. Although adopting visual metaphors in SG design is in its infancy, the SGs’ literature suggests they can improve engagement and facilitate learning of the intended teaching points [28,32,33]. However, SGs are considered complex, and they need to be designed while taking into consideration the limits of human cognitive capacity [34]. Cognitive Load Theory (CLT) offers a wide range of instructional design principles [35]. CLT has been used in the design of SGs to manage the limited cognitive capacity of the learner [36], and it highlights the limited capacity of working memory (WM) to process from 3 to 7 items at any given time, compared with the potentially unlimited capacity of long-term memory [37]. CLT aims to reduce the cognitive load imposed by instructional design (extraneous load) on WM to improve learning (germane load). CLT design principles mostly target the extraneous cognitive load imposed by poorly designed material [38,39]. These design principles include using worked examples and minimizing redundant information and split attention [38]. The aim of this study was to evaluate the impact of an SG on learning HTC in medical students during their first clinical exposure. To our knowledge, this is the first visual metaphor–enhanced SG implemented for teaching HTC to medical students. To evaluate this SG’s impact, Kirkpatrick’s training evaluation model was adopted, as it is the most widely used method in evaluating training programs [40] and is commonly used to assess medical educational interventions [41-43]. Kirkpatrick’s model was modified by Freeth et al [43] and adopted by the Best Evidence Medical Education Collaboration to assess medical teaching interventions [44]. The domains included are the following: affective (cognitive load, material difficulty level, and perceptions and satisfaction), cognitive (knowledge gains), and behavioral attitudes (objective structured clinical exam). The fourth level in Kirkpatrick ‘s model (outcomes related to the impact of the interventions on patients’ care) is not commonly measured in educational settings [45] and is beyond the scope of this study.

## Methods

### Overview

This pilot study used a mixed methods approach by combining a repeated-measures nonrandomized quasi-experimental design with a qualitative component. The study was approved by University of Auckland Human Participants Ethics Committee, reference number 015567. Med Metaphoria game design and development were funded by The New Zealand Health Innovation Hub, in collaboration with Counties Manukau District Health Board, who provided the first author with a clinical fellowship in Health Systems Innovation and Improvement.

### Recruitment

The University of Auckland has a 6-year undergraduate medical program, predominantly comprising basic sciences (Years 1-3) and then clinical practice (Years 4-6). Our sample was a preclinical population of year-3 medical students at the South Auckland Clinical Campus of the University of Auckland, who were attending a 10-week clinical methods module at the end of 2015. This is a foundation course for teaching clinical skills. All 83 students on the rotation were invited to participate in this study. Recruitment strategies involved both verbal and poster invitations. Students were preallocated, at the medical school administration level, to 1 of 2 teaching days (Wednesday or Thursday), independent of the investigators.

### Interventions

**Figure 3:**
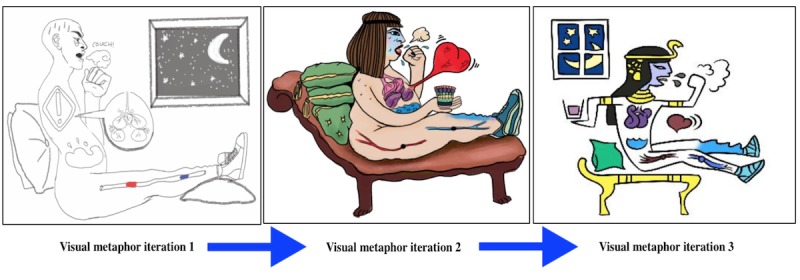
Iterative visual metaphor design.

To minimize potential contamination, the control group (PDF file group) was allocated to Wednesday, followed by the intervention group (Visual Metaphor Game; VMG group) on Thursday. This was done to minimize contamination in keeping with medical education recommendations [46], which suggested restricting access to the intended educational intervention. Both groups had the same face-to-face cardiac history teaching (whole morning session) delivered on the day of the interventions, followed by a one-off 40-min session, using either a PDF file or the VMG. Both groups used iPads to access content, whereby the same content was covered but differed in instructional design, as the PDF file was textual, and the game used visual metaphors. A visual metaphor–enhanced game (Med Metaphoria) was developed as a 3D SG that contains elements of HTC (Figure 1 and Multimedia Appendix 1). The 3D game was codesigned by the lead author in collaboration with clinicians, medical educationalists, code developers, and students. We followed a participatory iterative agile game development process [47], using the I’s development framework, proposed by Annetta et al for the design of SGs [48] (Figure 2). This “I’s” framework included the following elements: Identity, Immersion, Interactivity, Increasing complexity, Informed teaching, and Instructional. Game development (versions 1 and 2) incorporated student and clinician feedback by using a think-aloud protocol, originally developed by Ericsson and Simon [47,49] and later adopted by SGs’ design literature [50] until the final iteration (version 3) was developed (Figure 3). Although there are no significant differences between 2D and 3D educational games in terms of learning gains [51], 3D design was chosen as the main game design modality, despite a higher associated cost for the following reasons. First, a key teaching point in the history-taking visual metaphor is to avoid “tunnel vision” history-taking, which is similar to looking at a 2D pyramid, focusing on 1 causative side or system and ignoring the other 3D pyramid sides and systems. Therefore, 3D depth of the visual metaphor was essential to convey this point, which is commonly used in the game literature and called “spatial metaphor” [52]. Second, the literature has shown that health sciences students’ preference for 3D over 2D games [53]. Finally, the game literature has found that 3D games facilitate a more enhanced sense of presence than 2D games [53]. Overall, 2D graphics were also used if there was no spatial need for metaphors.

**Figure:**
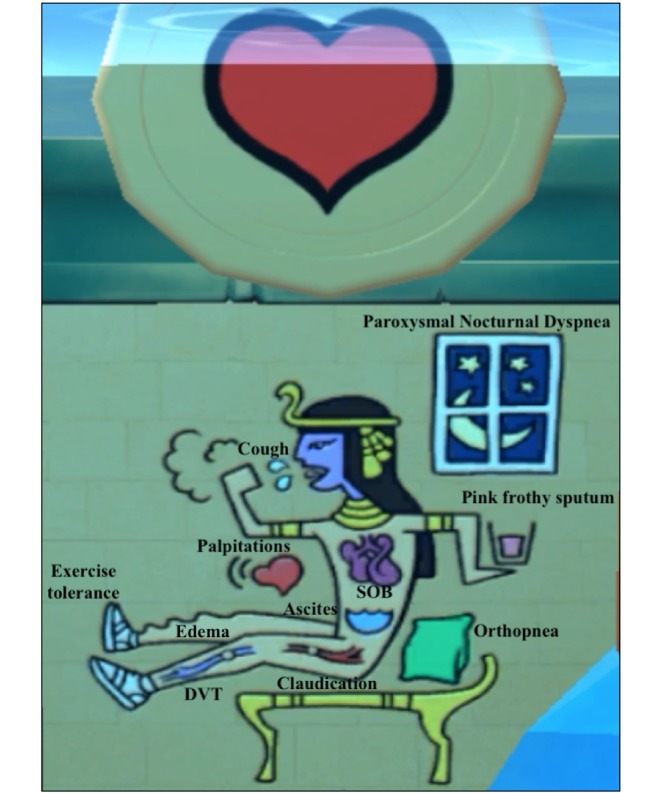
In-game snapshot showing the symptoms and signs relevant to the cardiovascular system.

**Figure:**
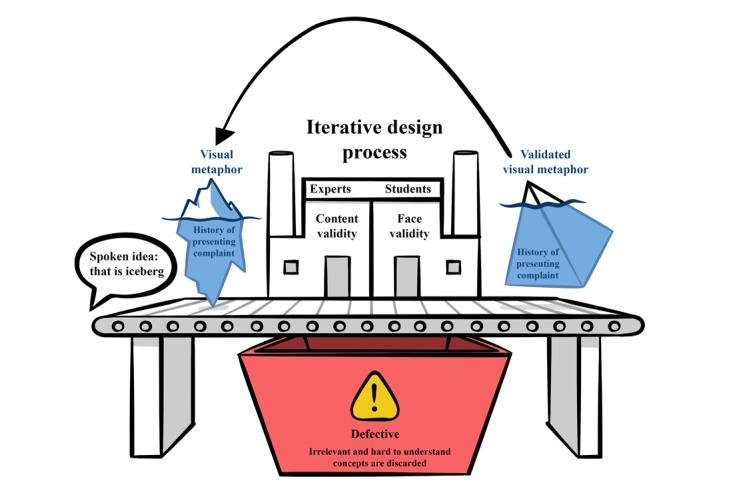
Visual metaphor and game design process in each game version.

The main characters in the game were the medical students in the submarine (the rescuer and students had the option of choosing their own personalized name) and John the Archaeologist (the patient). The game was a point-and-click adventure game, taking place in a 3D fantasy world where John was inadvertently locked up in a pyramid after deciphering a set of visual metaphors wrongly. The game rules included deciphering the visual metaphors correctly by associating them with the correct multiple-choice answer to rescue John, who is complaining of chest pain. The quicker this was done, the higher the score and rank achieved. Each section of the history taking was a separate part of the game journey. Audio visual and textual feedback was provided, in addition to static and animated game narrative and cut scenes. Students had to decipher the visual metaphors by choosing the right answer out of 3 or 4 multiple-choice answers per question. Although question order was constant, answer positions were randomly ordered for each question. The game had 3 difficulty levels, with the beginner level allowing 30 seconds per question, using the full textual question, followed by the intermediate level, which allows 20 seconds, with questions partially visible, and the advanced level, allowing 10 seconds, which only shows the visual item with no textual question. The total number of seconds per difficulty level was divided into thirds, and students were ranked into 1 of 3 ranks on the basis of their time to answer each question correctly: Consultant (first third), Resident (second third), or Intern (last third). Students also scored points corresponding to their speed in choosing the right answer (Figure 4).

**Figure:**
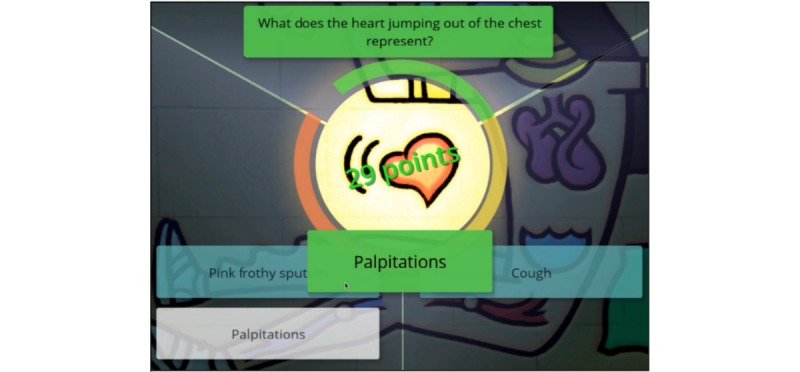
In-game snapshot showing the visual metaphor in the background, question and multiple choice options. The number of points (29) represents that it took the player in the beginner level one second to decipher the visual metaphor and answer the question correctly (out of the 30 seconds allocated per question).

### Development and Validation of Teaching Interventions and History-Taking Content Measures

Both PDF files and the VMG were developed after consulting with the recommended reading list the students received, which included a clinical skills module booklet and a recommended clinical skills textbook [54]. Content validity and instructional design of both interventions were carried out through iterative consultation with multiple junior and senior doctors, from medical and surgical disciplines, including Internal Medicine, Cardiology, Surgery, and Psychiatry. Face validity was established through iterative discussions with multiple medical students across the medical school program.

### Measures

For both groups, knowledge about HTC was assessed using a paper-based context-rich open-ended question. This was asked immediately before intervention (baseline), immediately after intervention (postintervention), and then again 7 weeks after intervention (follow-up). These tests were designed to assess baseline HTC knowledge and immediate and delayed recall (see Figure 5 for study design). This open-ended question was deemed more appropriate than using closed multiple-choice questions [55]. It read “what are the cardiac symptoms you would ask about as part of the history of presenting complaint in a patient presenting with chest pain?” The model answer template had a total of 25 symptoms. Answers that included lay or medical terms were accepted if they were consistent with the symptom meaning. Marking was conducted by a senior cardiology registrar, who was blinded to both groups, out of a total score of 25 based on predefined marking criteria (Multimedia Appendix 2).

**Figure:**
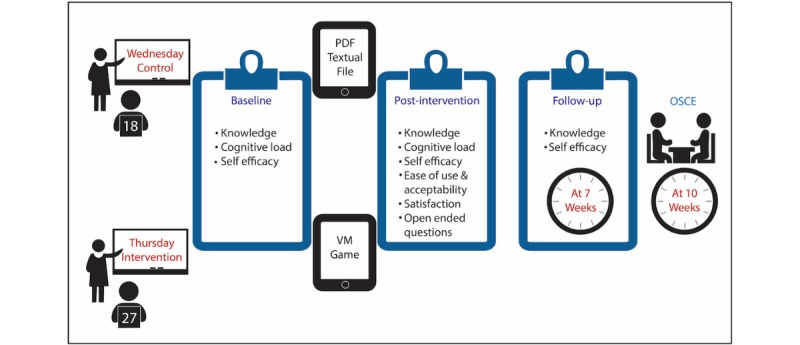
Study design and sampling time points.

Pre, post, and follow-up questionnaires were designed to assess the 3 levels of Kirkpatrick’s training evaluation model. These key points about the measures used are summarized in Table 1. The Mental Effort Scale [56] was used to assess students’ perceived history-taking cognitive load, whereas history-taking difficulty was measured by using the Difficulty Level Scale [57]. The Mental Effort Scale is a subjective rating scale for cognitive load, with a Cronbach coefficient alpha of .90 (internal reliability) [56]. This scale has been studied widely in the literature, and it has shown good psychometric properties [37]. It has been shown to be comparable to objective physiological measures and has good validity and reliability [58], and it is more sensitive and simpler than objective physiologic measures [37]. The adapted 5-point Likert scale is commonly used to assess cognitive load [57], where the question “In taking a history, I invest...” is answered on a 5-point Likert scale. As a one-off administration of the scale was shown to over or underestimate scores [59,60], it was administered at 2 timelines (baseline and postintervention). The perceived task difficulty level was also measured, as it is an important but different construct to cognitive load [61]. According to Gog and Paas, tasks perceived to be highly difficult might lead to using low mental effort in solving them [61]. This scale was administered to answer the question “I experienced history-taking as...”

Despite the known criticism of single-item scales [62], such as cognitive load and perceived task difficulty scales, research has shown these scales to have comparable properties with multi-item scales [63]. The Self-Efficacy Scale [64] was used to measure student perceived self-confidence in history taking. Medical students’ self-efficacy has been shown to influence academic performance [65-67]. Bandura’s seminal work on self-efficacy theory highlighted that competency to perform a certain task is not limited to only knowledge and skill acquisition but also in how the participant believes in their efficacy of reaching this goal [68]. In the context of SGs, Gee highlighted how they could increase self-efficacy [69]. The General Self-Efficacy (GSE) Scale devised by Schwarzer and Jerusalem [64] was used to measure student perceived self-confidence in history taking. GSE refers to the global confidence in one’s coping ability across multiple situations [64]. This scale has been used extensively in research, with an internal consistency of 0.75 to 0.91, with a reliability of 0.67 [70]. However, it is considered a nondomain-specific scale, which goes against Bandura’s domain-specific conceptualization of self-efficacy [71]. Therefore, GSE scale adaptation was necessary to make it history-taking domain specific to fit with Bandura’s self-efficacy theory [71]. Given that self-efficacy is dynamic, researchers have advised to measure this construct longitudinally rather than cross-sectionally [72]. Although some researchers have measured self-efficacy at 2 time points, medical educationalists have recommended doing so more than twice [71]. Given that both educational interventions were delivered using a technologically enhanced medium (an iPad) in this study, the Technology Acceptance Model (TAM) was used [73]. The TAM’s Ease of Use and Acceptability questionnaire has been previously used to investigate medical students’ [74] and physicians’ intentions to use technology [75,76]. Owing to its robustness, simplicity, and adaptive nature, it has become one of the most widely used models in measuring technology acceptance [77].

SGs that involve instructional design optimization have been shown to improve satisfaction levels [78]. Therefore, a satisfaction questionnaire was designed as it could serve as an indirect academic performance measure [79]. These questions were drawn from other relevant medical education literature evaluating technologically enhanced educational interventions [80,81]. The questionnaire face and content validation process followed an iterative design process similar to game design (Figure 2). Immediately after the intervention, 3 open-ended questions were used to assess students’ perceptions and suggestions of what they learned, enjoyed, and did not enjoy, as well as suggestions for further improvement.

History-taking clinical skills were objectively assessed at 10 weeks postintervention by comparing end-of-year objective structured clinical examination (OSCE) results for the history-taking station. The history-taking station was designed independent of the investigators, and it targeted assessment of abdominal pain; however, the overall history-taking structure and pain questions were similar.

**Table 1:** Measures used and relevant information.

Scale name and Construct		Items	1 is	5 is	Timelines
**Mental effort**					
	Cognitive load	1	Very low	Very high	Baseline and postintervention
**Perceived difficulty**					
	Task difficulty	1	Not difficult at all	Very high difficulty	Baseline and postintervention
**General Self-Efficacy**					
	Self-confidence in history taking	10	Strongly disagree	Strongly agree	Baseline, postintervention, and 7 weeks later
**Technology acceptance model**					
	Ease of use	6	Strongly disagree	Strongly agree	Postintervention
	Usefulness	6	Strongly disagree	Strongly agree	Postintervention
**Satisfaction**					
	Satisfaction	8	Strongly disagree	Strongly agree	Postintervention
**Knowledge**					
	Open-ended knowledge question	Marks out of 25	—^a^	—	Baseline and postintervention
**Clinical skills**					
	Objective structured clinical examination	Marks out of 25	—	—	10 weeks postintervention

^a^Not applicable.

### Statistical Analysis

Descriptive statistics were used to assess baseline demographic characteristics, and mean scores of continuous variables were calculated. Chi-square or Fisher exact tests were used to test for associations between the PDF and VMG groups. To examine changes in self-efficacy and knowledge across baseline, postintervention, and follow-up, a repeated measure analysis of variance was carried out. This was adjusted for confounders, including age, gender, ethnicity, education level, marital status, and specialty preference. Interaction between group and time were also tested and adjusted in the model if it was deemed to be significant (*P*<.05). All the analyses were carried out using SAS (version 9.3; SAS Institute) software. Arbitrary missing data at the follow-up test were handled using multiple imputation, using the 2-fold fully conditional specification approach proposed by Welch et al, using the predicted mean matching [82].

### Qualitative Analysis

Data analysis was conducted using the 6-phase thematic analyses guide developed by Braun and Clark [83].

Authors HA and MA independently followed these 6 steps, and consensus was reached using the constant comparison approach where disagreements were discussed until mutual agreement was reached. The first phase involved entering responses in an Excel spreadsheet where they were read and reread as part of the data familiarization stage, and notes were taken for noticeable patterns. Manual coding was performed through highlighting repeated meanings that represented certain patterns as part of a data driven analysis. After data were coded, broader themes were generated. We used tables of codes and generated themes accordingly. At this stage, themes were reviewed and refined against the coded data level, followed by the whole dataset level. These themes were refined and named to ensure they reflected the meaning of the dataset they represent, highlighting interesting points and providing any potential explanations. Each theme was analyzed separately and in relation to other themes within the overall dataset meaning. Finally, report writing involved writing the meaning of the datasets on the basis of the generated themes supported by data extracts. At this stage, critical explanations about the rationale for such themes in the context of our research question about learning enhancement were sought.

## Results

### Sample Characteristics

A total of 83 students were eligible for the study, and 46 students agreed to participate (55% response rate). In total, 1 student left the PDF group, leaving 18 participants in this group and 27 in the VMG group. The student demographic characteristics in both groups were similar, including age, gender, specialty preference, and gaming frequency (Table 1). All students participated in the baseline and postintervention measures. Of those, 24 (88%) of the students in the VMG group and 14 (73%) students in the PDF group completed the follow-up test 7 weeks later. The sample characteristics are provided in Table 2.

**Table 2:** Sample characteristics.

Baseline characteristics		Group		Total	*P* value
		PDF	Game		
**Gender, n (%)**				.73^a^
	Male	4 (21.1)	8 (29.6)	12 (26.1)	
	Female	15 (79)	19 (70.4)	34 (73.9)	
**Age (years), n (%)**					.68^a^
	19-21	10 (52.6)	18 (66.7)	28 (60.9)	
	22-25	8 (42.1)	8 (29.6)	16 (34.8)	
	>26	1 (5.3)	1 (3.7)	2 (4.4)	
**Ethnicity, n (%)**					.32^a^
	NZ European	5 (26.3)	8 (30.8)	13 (28.9)	
	Maori	2 (10.5)	3 (11.5)	5 (11.1)	
	Pacific	5 (26.3)	2 (7.7)	7 (15.6)	
	Asian	1 (5.3)	6 (23.1)	7 (15.6)	
	Other	6 (31.6)	7 (26.9)	13 (28.9)	
**Marital status, n (%)**					.58^a^
	Single	16 (84.2)	18 (69.2)	34 (75.6)	
	Couple/De Facto	3 (15.8)	7 (26.9)	10 (22.2)	
	Married	0	1 (3.9)	1 (2.2)	
**Specialty, n (%)**					.75^a^
	Medicine	10 (52.6)	14 (53.9)	24 (53.3)	
	Surgery	0	2 (7.7)	2 (4.4)	
	Subspecialities	2 (10.5)	3 (11.5)	5 (11.1)	
	Do not know	7 (36.8)	7 (26.9)	14 (31.1)	
**Gaming frequency, n (%)**					.88^a^
	Once a day	1 (5.3)	3 (11.5)	4 (8.9)	
	More than one a day	1 (5.3)	2 (7.7)	3 (6.7)	
	Once a week	3 (15.8)	5 (19.2)	8 (17.8)	
	More than once a week	0	1 (3.9)	1 (2.2)	
	I do not play games	14 (73.7)	15 (57.7)	29 (64.4)	
**Education, n (%)**					.31^b^
	Undergraduate entry	12 (63.2)	20 (76.9)	32 (71.1)	
	Master’s degree	7 (36.8)	6 (23.1)	13 (28.9)	
**Enrolment status, n (%)**					.50^a^
	Domestic	19 (100)	25 (92.6)	44 (95.7)	
	International	0	1 (7.4)	2 (4.4)	

^a^Fisher exact test used.

^b^Chi-square test used.

### Knowledge and Self-Efficacy

**Figure 6:**
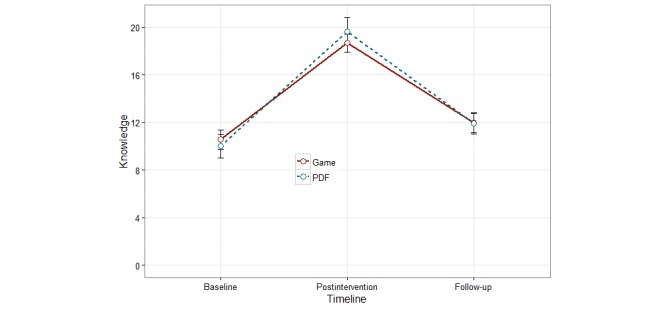
Knowledge across baseline, postintervention and follow up time points (no statistically significant differences).

**Figure 7:**
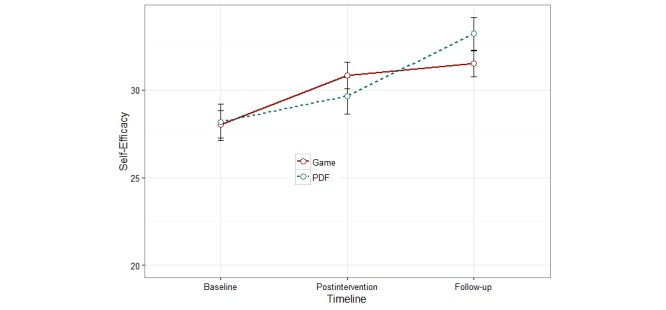
History-taking self-efficacy across baseline, postintervention and follow up time points (no statistically significant differences).

Although between-group differences in knowledge and self-efficacy at postintervention and 7-week time points compared with baseline were not significant (knowledge *P*=.95 and self-efficacy *P*=.85), the within-group differences were statistically significant (*P*<.001) for both groups (Figures 6 and 7). This significant difference persisted across unadjusted and adjusted models (adjusted by age, gender, ethnicity, education level, marital status, and specialty preference).

### Clinical skills (Objective Structured Clinical Examination Scores)

The OSCE took place 10 weeks after the intervention. No significant differences between the intervention and control groups were found (*P*=.60; Table 3).

**Table 3:** History-taking cognitive load, difficulty level, objective structured clinical examination results, ease of use, usefulness, and satisfaction means, SD, and P values.

Outcome, group, and timeline			Mean (SD)	*P* value
**Cognitive load**				.88^a^
	**PDF**				
		Baseline	3.94 (0.64)	
		Post	3.89 (0.68)	
	**Game**				
		Baseline	3.81 (0.62)	
		Post	3.74 (0.66)	
**Difficulty level**				.68^a^
	**PDF**				
		Baseline	2.94 (0.73)	
		Post	3.06 (0.64)	
	**Game**				
		Baseline	2.96 (0.59)	
		Post	3.04 (0.52)	
**Satisfaction**				<.001
	**PDF**			
		Post	23.72 (4.35)	
	**Game**			
		Post	29.85 (4.86)	
**Usefulness**				.30
	**PDF**			
		Post	21.39 (4.1)	
	**Game**			
		Post	22.59 (3.62)	
**Ease of use**				.19
	**PDF**			
		Post	23.28 (3.68)	
	**Game**			
		Post	24.7 (3.36)	
**Objective structured clinical examination**				.60
	**PDF**			
		Post	14.33 (4.52)	
	**Game**			
		Post	15.04 (4.28)	

^a^Wilcoxon 2-sample test (nonparametric test).

### History-Taking Cognitive Load and Level of Difficulty

There were no statistically significant differences from baseline between the 2 groups in either cognitive load scores (*P*=.88) or level of difficulty scores (*P*=.68; Table 2). Similarly, the ease-of-use and usefulness levels assessed at postintervention did not reach statistical significance (ease of use *P*=.19 and usefulness (*P*=.30; Table 3).

### Satisfaction

There was a significant difference in satisfaction levels between the groups, with the VMG group having a statistically higher satisfaction level compared with the PDF group (mean difference of 6.13; 95% CI 3.27-9.00, *P*<.001; Table 3).

**Qualitative Results**

The overall findings indicate that the VMG and PDF file presented both HTC and structure well to participants. However, although VMG was viewed as a learning tool that aided understanding, encoding, and recalling history taking in an engaging and fun way, the PDF file was viewed as easy to read but difficult to learn or remember. In addition, the PDF file was seen as an electronic handout, which was no different to other handouts that were boring and nonengaging. A total of 4 major themes emerged from this analysis for both interventions: interventions as learning tools, enjoyment and engagement, generalizability, and suggestions for future improvement (Table 4). Identities are masked and replaced by pseudonyms in the section below to preserve confidentiality.

**Table 4:** Thematic analysis summary.

Game	Shared main themes	PDF
Memory aid for conceptual, procedural, and metacognitive knowledge development	Learning tool	An easy-to-read but hard-to-learn reference tool
Enhances enjoyment in learning history taking	Affects enjoyment and engagement	Neither engaging nor enjoyable
Text size, spelling, grammar optimization, add skip button, and increase difficulty levels	Design and functionality optimization suggestions	Add pictures, color and animations, as well as enhancing interactivity
Game specific theme: Game is generalizable to other clinical scenarios of different systems	—^a^	—

^a^Not applicable.

### Theme 1: Interventions as Learning Tools

#### Visual Metaphor Game as a Memory Aid for Conceptual, Procedural, and Metacognitive Knowledge Development

A recognized benefit of using visual metaphors is enhancing memory retrieval through activating prior knowledge, thus allowing new knowledge and concepts to be learned [21]. For example, Stacey discussed how visual metaphors enhanced understanding and recall: “The use of symbols made important factors to ask about easy to recall.” Michael described how visual metaphors went beyond being just a memory aid to facilitating the “how” aspects of knowledge in terms of structure and systematic history-taking approach, as they added a “very good systematic process with visual symbols – aids memory,” whereas James shared that visual metaphors “helped realise that a system is important to follow - logical order/sequence.” On a metacognitive level in terms of “thinking about thinking” [84], visual metaphors enable the interpreter to make strong associations among concepts [85]. This enables the students to understand how they think and process information. Tanya stated that she enjoyed “the metaphors - since it has visual association with aspects of history-taking. I like that it is very structured in its approach,” and Sarah highlighted how visual metaphors were “very effective associating concepts with images.”

#### PDF Viewed as an Easy-to-Read but Hard-to-Learn Reference Tool

Although a majority of the PDF group participants viewed it as a clear and concise list, they found it hard to learn and remember. Although it was seen by William as “easy to read bullet points and concise information,” it was “not interactive, cannot remember just by reading, boring.” Perceiving the PDF file as a reference tool is consistent with the medical education literature, as technology-based handouts were thought of as a reference tool [86,87]. The negative impact of lack of PDF file interactivity on learning outcomes has been observed in a previous study where students in the noninteractive PDF handouts group had significantly reduced educational outcomes compared with other interactive electronic handouts [88]. This may have implications for medical education in general, as information (including difficult to understand concepts) is often presented in textual format, without accompanying images.

### Theme 2: Instructional Design Influences Enjoyment and Engagement

#### Visual Metaphors Enhance Enjoyment in Learning History Taking

Almost all the VMG participants enjoyed the VMG. For example, Jake enjoyed “the results afterwards, i.e., remembering content of history-taking.” Stacey highlighted this by stating:

I enjoyed how easy it was to use…..made things fun to follow.

This highlighted how enjoyment was linked to improved learning the material, as well as the ease of playing the game. This increase in game enjoyment is reported in the SGs literature, as studies have highlighted how students experienced high enjoyment levels while playing SGs [28,89]. Some researchers explained this increased enjoyment by the balance achieved between the learner’s abilities and the challenge posed by the game [90], which is consistent with Stacey’s statement above.

#### PDF Was Neither Engaging Nor Enjoyable

A majority of the PDF group participants found it boring and nonengaging because of a lack of visuals or interactivity, as Josephine stated, “It was not interactive at all, so it was not fun and I probably won't remember the stuff well.” Sam mentioned that “It was plain and was not necessarily enjoyable but just another handout but electronic.” These observations are supported by the literature, as technologically noninteractive modalities of presenting information, such as textual information, are considered less productive than interactive educational modalities [91]. Incorporating visual and verbal modalities of information is said to improve student understanding of taught material [92], which could explain the above statements.

### Theme 3: Visual Metaphor Game Is generalizable to Other Clinical Scenarios of Different Systems

Overall, a majority of the VMG group participants appreciated the teaching value of the VMG and suggested generalizing it to teach other organ systems and clinical scenarios. Martin suggested the following:

To have different cases played out but with the same format so we learn about other systems with depth too. I feel that it has definitely helped me a lot with my memory recall, thanks.

Mike said the following:

I think the game is focused on one type of clinical problem, chest pain. It would be nice to include more scenarios

This did not feature in the PDF group responses. Visual metaphor use with games has been reported to serve as an “anchor” for conceptual knowledge [28], and this could explain the above responses. In the current study, though the history-taking visual metaphor had a cardiac focus, this could be easily applied to other organ systems (eg, respiratory system).

### Theme 4: Design and Functionality Suggestions for Improvement

Participants in both groups offered suggestions on how both interventions could be optimized. Although PDF group participants offered several design suggestions, the VMG group had limited design suggestions.

#### Game Prototype Design and Functionality Suggestions

VMG participants made suggestions as to how the functionality and user interface could be optimized. In terms of design, some participants suggested text size optimization, as Simon commented “make text larger maybe?” Another suggested enlarging the buttons’ size. Others suggested spelling and grammatical corrections and adding more levels of difficulty. In terms of functionality, suggestions included adding back and skip buttons, lag improvement, and minimizing repetition. Some participants, such as Harris, commented on the game prototype lagging and requested to “make it faster if possible. Great concept.” Stephanie shared a similar opinion that we need to “Make the game respond faster.” Lag in the game literature refers to the game delay in response to the actions of the player [93], and this has been shown to negatively impact the gameplay experience [49,93,94]. A possible explanation for the reported lag is the use of data-rich 3D graphics in the game [95].

Angela appreciated the positive impact of repetition on memory and acknowledged that repetition was less enjoyable when they have learned the material. This was captured in her response: “repetition was really good for memory, but after I was able to remember it, it became a little boring, sorry.” The use of repetition in SGs is associated with improved learning [96], and the reported boredom after learning content is a good illustration of the flow state proposed by Csikszentmihalyi [95]. In the game literature, a balance needs to be struck between the game task difficulty and the player’s ability to reach a state of flow [97]. On the basis of this, hard games could frustrate the player, whereas easy ones could bore the player. Therefore, the literature suggests increasing game difficulty as the user’s knowledge improves, to maintain this state of flow. This increase in difficulty levels was suggested by Jasmin, as she wanted “an even more advanced level.” However, others enjoyed the current 3 difficulty levels, which is evident in Yvonne’s response about enjoying the “different levels to eventually learn history from memory.” Finally, given that the reported boredom only occurred after the material was learned, which is the educational purpose of this SG; adding more difficulty levels might be questionable.

#### PDF Design and Functionality Optimization Suggestions

Most PDF group participants had suggestions to enhance PDF design and functionality through adding pictures, color, and animations, as well as enhancing interactivity.

In terms of design, Patrick suggested “using more animations, colours, pictures making it more interesting and interactive,” whereas Patricia suggested adding functions, such as a record button, and a function to write on the file by having a “marker so you can write on and interact with the file to improve memorisation,” and Ronald suggested to “have a record button that records the conversation and puts all patients’ responses into each section immediately.” These suggestions are in line with educational research, which showed that interactive audio visual functionalities enhance learning outcomes [98].

## Discussion

### Summary of Key Findings in This Study

This quasi-experimental pilot study that utilized both the quantitative and qualitative research methods approach evaluated a visual metaphor–enhanced 3D SG in teaching cardiac HTC to year-3 medical students in comparison to PDF-delivered teaching. More than half the sample indicated a preference to specialize in General Medicine in the future and were not playing games regularly. This study showed that the game is comparable to and as effective as textual information in increasing knowledge gains, enabling self-efficacy, and managing cognitive load, level of difficulty, perceived ease of use, and acceptability at various time points. However, the game was superior to textual information with regard to higher satisfaction scores relative to the PDF group. The reasons for this could be drawn from the qualitative analyses, which showed that the VMG was perceived to be a useful visual memory aid, more enjoyable, and transferrable to other clinical scenarios. On the contrary, the PDF group found the PDF file to be boring and merely another handout that was neither interactive nor engaging.

### Quantitative Findings in the Context of the Literature

The quantitative findings were consistent with the current pre and postgraduate medical education SGs literature, which shows that they are at least as effective as traditional teaching methods. Consistent with the higher satisfaction scores in the VMG group and the qualitative findings of students viewing the game as more enjoyable, fun, and as a useful memory aid, 2 systematic reviews reported that SGs have the advantage of increasing students’ enjoyment and interest in the topic taught [99,100]. The improvement in satisfaction was consistent with medical education game use among medical students when compared with traditional teaching [81,101]. The main benefit of the new teaching approach was that the VMG was statistically more significant in terms of satisfaction and was more favored in the qualitative analysis. As student satisfaction improvement is on its own an important learning outcome that complements academic achievement [102], this significant satisfaction improvement in the game group is worthy of further consideration. Research has shown that improved satisfaction enhances student academic performance, and its enhancement is strongly advised by educationalists [103,104]. A previous systematic review assessing the impact of educational games on medical students’ learning outcomes found potential for improving learning outcomes, but it highlighted the need for more rigorous research [105]. Graafland et al systematically reviewed the literature for the impact of SGs on training health professionals and assessed their validity [106]. They included a total of 30 SGs, 17 of which were designed for educational purposes. However, none of these games were fully validated. The authors suggested validating games before incorporating them into teaching, and such validation needs to include content, face, and concurrent and predictive validity. In 2013, a Cochrane review looking at the use of games as a teaching method for health professionals found insufficient evidence for or against their use [107]. Wang et al conducted a systematic review in 2016 of SGs in medical education, and they identified 42 studies reflecting an increase in the number of SGs. Of these, only 19 studies included an evaluation of SGs, with a majority of these (n=17) associated with significant educational benefits [108]. Out of the 19 studies, only 4 were relevant to a medical student population, which assessed the use of SGs on medical students’ knowledge and skill acquisition [81,109-111]. Only 1 of these studies found a significant improvement in knowledge, but this was for immediate recall only, as long-term retention was not assessed [111]. There are several possible reasons why this study showed no significant quantitative differences between the teaching methods apart from satisfaction scores. The duration (40 min) for learning the textual information may be a confounder, as it was regarded as too long by many students, which meant that students started interacting and assessing each other. As assessment drives learning [112], it could be argued that the PDF group assessing each other instead of engaging with the iPad only could have improved their scores independent of the PDF file. The influence of student-student interaction has been found in previous work to be similar to interactions between students and facilitators [81]. SGs are usually played more than once, which is different to the one-off 40-min play session design in this study, and this could also explain the lack of significant findings. In 2013, a meta-analysis of SGs reported how a lack of significant difference between games and traditional teaching could be attributed to the lack of playing the game on multiple occasions, as normally happens [32]. In that study, compared with multiple gameplay sessions, single session gameplay was not more beneficial than traditional teaching methods. Another possible reason for lack of significant difference in scores between the groups is the progression of skill development over time in the absence of formal teaching [3]. Although the VMG group only had 40 min of gameplay, both groups had unlimited access to traditional teaching material from which the PDF file was designed, which could have been a confounding factor in this study. Another possible explanation for this result is that the control group’s awareness of its control status, as text PDF file is 1 of 2 interventions where the other is a game, influenced the group’s behavior, as the members of the group may have tried to outperform the intervention group. This is a statistical confounder called the John Henry Effect [113], evident by PDF group participants assessing each other during the experiment and highlighted by the fact that a participant left the control group as soon as she knew she saw the PDF file was not the game. Embedding qualitative research alongside quantitative approaches has been suggested as a way of addressing the John Henry Effect [114].

### Qualitative Findings in Context of the Literature

The qualitative research in this study was to investigate students’ perceptions about the 2 educational approaches. Although both were seen as learning tools, the depth of such learning was better in the VMG group (conceptual, procedural, and metacognitive knowledge levels) compared with the PDF group (conceptual level). Several students touched on educational benefits of visual metaphors, for example, students saw how it enabled them to “associate” visual metaphors with HTCs. This mirrors findings on how visual metaphors work in terms of associating existing knowledge of certain visuals (familiar objects) and linking them with new (unfamiliar) concepts [20,115-117]. This associative function could be because of its visual nature, which, according to Pavio’s Dual Coding Theory, creates cognitive associations with textual information, thus enhancing learning [118]. Active cognitive processing marrying new-to-previous knowledge is commonly referred to as “meaningful learning,” a term that was pioneered by the educational psychologist, Ausubel, in the 1960s. He stated that “If I had to reduce all of educational psychology to just one principle, I would say this: The most important single factor influencing learning is what the learner already knows. Ascertain this and teach him accordingly” [119].

The VMG group’s awareness of such associative cognitive processes shows how visual metaphor enabled them to “think about thinking,” which is an important metacognitive skill that is essential for self-regulated learning [120]. Another benefit reported by students using visual metaphor was reaching a personalized deeper meaning of history taking. This “Cognitive Elaboration” [117] was observed in some responses alluding to reaching a conceptual understanding of how history taking has multiple interrelated parts, which need to be uncovered systematically and carefully. This was consistent with the visual metaphor literature, as it was seen to provide the learner with a deeper meaning and understanding of the presented material [115].

### Game Design Considerations

For the VMG group, most participants suggested developing the game for other organ systems and clinical scenarios suggesting the usefulness of the VMG. Few students commented on the need to address the gameplay lag. This need for immediacy is one of the main features contributing to improved gameplay [121], and this will be addressed in the next iteration of the game. In terms of implications for this study, clinical educational interventions that focus on HTC should continue to explore the value of visual metaphor use delivered through SGs to better teach this crucial skill. First, our SG was delivered as a one-off 40-min session, and according to the literature, more than one session of playing SGs yielded significantly better results compared with traditional teaching [32]. Therefore, the SG could be tested using a bigger sample and randomized study design for multiple sessions, to assess its actual impact compared with traditional teaching, given the overall literature support of SGs’ benefits. Second, students’ feedback on game optimization is being taken into consideration, as we are at the final stages of developing another game iteration, taking into account students’ feedback. It is hoped that this version will be tested in a multicenter randomized controlled trial to assess its impact against traditional teaching. Third, another area that warrants further exploration is the use of visual metaphors in teaching other clinical topics, as students’ qualitative feedback showed its perceived generalizability to other history-taking topics apart from cardiac history taking. In terms of cross-cultural impact, SGs could be useful for medical students across the globe, especially with the technically savvy current and future millennial medical students. Although most of the developed SGs are from developed countries, it is expected that other less-developed countries will follow suit [30]. The global benefits of SGs are widely recognized [122], as once they are developed and deployed, they can be accessed from all around the world, and collaboration could be sought to translate SGs to local languages to facilitate medical education efforts across the globe. This is increasingly made possible with higher access to the internet internationally. An example of this exponential increase to mobile phone access has jumped from 10% in 2002 to 80% in 2015 in Kenya, with more access to smartphones for higher education students [123]. Internet is seen by a majority of the 32 underdeveloped countries as beneficial for education [124].

### Limitations

This study has several limitations. First, this study included a relatively small sample size, but of note, this was a pilot study, targeting one of the medical school’s 3 main teaching sites in Auckland. The response rate was 55%, which is consistent with the SGs literature [125], as some studies response rates were as low as less than 50%. The studies with higher response rates incorporated the interventions as part of the curriculum, which was not possible in this study. Although a randomized study design would have been ideal, it was not feasible, as students were preallocated at the medical school administration level, factoring in students’ residential proximity to the clinical teaching campus. This lack of contextual feasibility has been previously cited as one of the barriers to randomized study design adoption in medical education [126,127], and this is reflected in that less than 20% of SGs studies have used a randomized study design [128]. Moreover, participants were not blinded, which is a common issue in educational interventions, such as games, as students can easily identify the intervention. In addition, student-student interaction in the PDF group was not possible to control, which could have influenced their learning; potentially reducing the time limit for the PDF and VMG groups from 40 min may have helped address this issue. Finally, our design included a one-off game session, which differs from the usual gameplay experience of playing it more than once, which has been found to explain the lack of positive findings associated with SGs when compared with traditional teaching methods [32].

### Conclusions

In a mixed-method experimental pilot study, we provide evidence that 40 min of playing an SG is as effective as textual information in teaching cardiac history taking to year-3 medical students, with the added value of increasing student satisfaction compared with traditional teaching. The qualitative analysis showed the game was more engaging, fun, enjoyable, and perceived as a useful visual memory aid. The game was as effective for the 3 Kirkpatrick model levels of evaluating educational interventions: affective, cognitive, and behavioral attitudes. Although SGs are in their infancy, their potential educational benefits should be harnessed, considering the current and future digitally adept medical students, especially when motivation and enjoyment of learning is at stake.

## Appendix

Multimedia Appendix 1 A showreel of the Med Metaphoria game.

Multimedia Appendix 2 Knowledge test marking criteria.

## Abbreviations

CLT: cognitive load theory
GSE: General Self-Efficacy
HTC: history-taking content
HTP: history-taking process
OSCE: objective structured clinical examination
SG: serious game
TAM: Technology Acceptance Model
VMG: Visual Metaphor Game
WM: working memory
