# Supplementary material for: Impact of Using a 3D Visual Metaphor Serious Game to Teach History-Taking Content to Medical Students: Longitudinal Mixed Methods Pilot Study
Source: JMIR Serious Games. 2019 Sep 26;7(3):e13748. doi: 10.2196/13748 (PMC6788337; doi:10.2196/13748)
Supplement: Multimedia Appendix 2 [file games_v7i3e13748_app2.pdf]

Cardiac symptoms as part of the history of presenting complaint in a patient presenting with chest pain.

This includes the following: pain assessment, cardiac review of systems, relevant past medical history and cardiac risk factors.

Can mark any mention of symptoms whether in medical jargon or lay terms as long as the meaning is consistent with one of the 25 items below.

Marking is out of 25 and each item is scored a **maximum** of one mark each.

ie, if a student stated gradual onset, they get one mark but if the answer included all answers of item number 2, they still get one mark.

1. Site of maximum intensity
2. Onset and duration (when did it start, gradual or sudden in onset, when pain is present, how long does it last for?).
3. Character of the pain (sharp, dull, stabbing or throbbing)
4. Radiation to other sites, if so where
5. Associated symptoms (nausea, vomiting, sweating, fever etc)
6. Timing and frequency of pain (is it constant or intermittent)
7. Exacerbating factors (what makes it worse)
8. Alleviating factors (what makes it better)
9. Severity of pain (e.g. severity of pain on a scale from 1-10)
10. Swelling of the feet, ankles (Peripheral oedema)
11. Shortness of breath on rest or on exertion (dyspnoea)
12. Shortness of breath lying down (orthopnoea) relieved by sitting up/ extra pillows.
13. Shortness of breath causing the patient to wake up at night: relieved by sitting up/ extra pillows.
14. Awareness of irregular or rapid heartbeat (palpitations)
15. Swollen abdomen with weight gain (Ascites)
16. Pain in lower limbs on brisk walking (Intermittent claudication)
17. Pain in the calf muscle (Deep vein thrombosis)
18. Pink frothy sputum
19. How far can the patient walk (Exercise tolerance)
20. History of hypertension (HTN, high blood pressure, high BP)
21. History of hypercholesterolemia (dysplipidemia, high cholesterol)
22. History of diabetes
23. History of smoking
24. Family history of heart disease
25. History of alcohol consumption
